# A Rare Case of Macrophage Activation Syndrome Presenting as the First Manifestation of Systemic Lupus Erythematosus

**DOI:** 10.1177/2324709618812196

**Published:** 2018-11-15

**Authors:** Pooja Poudel, Thein Swe, Sheetal Rayancha

**Affiliations:** 1 State University of New York Upstate Medical University, Syracuse, NY, USA; 2 Interfaith Medical Center, New York, NY, USA

**Keywords:** MAS, SLE

## Abstract

Macrophage activation syndrome (MAS) itself is a rare, potentially life-threatening complication of a rheumatic disease, mostly seen in juvenile idiopathic arthritis. It infrequently occurs in systemic lupus erythematosus (SLE), and it is extremely rare to be the first presentation of SLE. In a study of 511 patients with SLE, 7 cases (1.4%) of MAS were identified. In all the cases, MAS was simultaneous to the presentation of SLE in this article, we report a case of a patient with MAS who presented with fever, rash, and high ferritin level up to 16911 ng/mL. A high degree of suspicion is required that high fever and rash can be clues to MAS. Early diagnosis is necessary since mortality rates remain high for untreated cases.

## Introduction

Macrophage activation syndrome (MAS) is a life-threatening condition. It belongs to the hemophagocytic lymphohistiocytosis (HLH) group of diseases, which includes familial HLH and secondary HLH. Secondary HLH is triggered by several causes that disrupt immune homeostasis, which includes infection, drugs, rheumatic disorder, and malignancy. HLH is characterized by proliferation and activation of T lymphocytes and macrophages, which causes an excessive inflammatory response and hypersecretion of cytokines. Clinically, patients usually present with prolonged fever, pancytopenia, hepatosplenomegaly, liver function abnormalities, hyperferritinemia, and coagulopathy.^1^ Systemic lupus erythematosus (SLE) is a systemic autoimmune disorder that involves multiple visceral organs. In adults, MAS is rarely associated with SLE^2^ the incidence of MAS associated with SLE is about 0.9% to 4.6%.^3,4^ In this article, we report a case of MAS that occurred as the first manifestation of SLE.

## Case Presentation

A previously healthy 26-year-old Caucasian woman was admitted due to high fever with rash for 2 days. The fever and rash started after lamotrigine was started for her bipolar disorder 1 week ago. However, on further questioning, she also had history of alopecia, arthritis, and oral ulcers intermittently. Her past medical history was unremarkable for rheumatic disease, severe infections, or immunodeficiency. Her family history was also negative for rheumatic disease. On admission, vital signs were normal except for the temperature of 101.5°F. On physical examination, she had diffuse erythematous maculopapular non-itchy rashes over her face and chest without mucocutaneous involvement. Since she complained of the rashes after starting the new medication, we initially treated her as an allergic reaction to the new drug with diphenhydramine and methylprednisolone. However, she continued to have fever spikes along with worsening of her rash.

**Figure 1.**
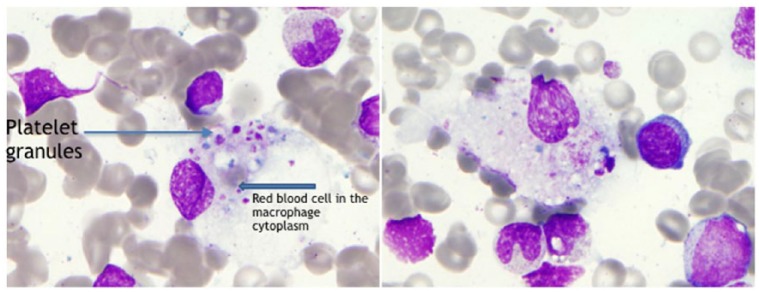
Slides from bone marrow biopsy showing hemophagocytosis.

Laboratory results showed white blood cells 1.7 × 10^9^/L, absolute neutrophils 1.51 × 10^3^/µL, absolute lymphocytes 0.08 × 10^3^/µL, hemoglobin 10.3 g/dL, platelets 138 000 µL, aspartate transaminase 57 U/L, alanine transaminase 19 U/L, triglycerides level 266 mg/dL, fibrinogen 273 mg/dL, ferritin level 16911 ng/mL (normal = 13-150 ng/mL), and elevated lactate dehydrogenase 1767 U/L. Immunological screening was positive for antinuclear antibody (ANA) homogeneous pattern 1250 (normal = 0-49 1/dilution), ANA speckled pattern 6250 (normal = 0-49 1/dilution), anti-double-stranded DNA antibody 344 IU/mL (normal = 0-99 IU/mL), anti-histone antibodies 210 AU/mL (normal = 0-99 AU/mL), serum C3 complement 35 mg/dL, serum C4 9 mg/dL, erythrocyte sedimentation rate 56 mm/h, and C-reactive protein 23.5 mg/L. Blood cultures, urinalysis, and chest X-ray were unremarkable. Viral panel testing for Epstein-Barr virus, cytomegalovirus, herpes virus, hepatitis B and C, HIV, and RPR (rapid plasma reagin) tests were unremarkable. Computed tomography scan of chest showed axillary, cervical, and supraclavicular lymphadenopathy. Since the patient had elevated ferritin and lymphadenopathy seen on computed tomography, there was a concern of MAS. Bone marrow biopsy showed an increased number of macrophages showing hemophagocytosis suggestive of MAS (Figure 1). Soluble IL-2 receptor was found to be high 3463 U/mL (normal = 223-710 U/mL), and CD 15/56 absolute natural killer (NK) cells was low 7 cells/µL (78-470 cells/µL). We also did axillary lymph node biopsy, which was negative for malignant lymphoproliferative disorder and it showed some hemophagocytosis.

The presence of fever, cytopenia, elevated ferritin, low NK cell activity, elevated soluble IL-2 receptor, and hemophagocytic cells in bone marrow and lymph node led to the main diagnosis of MAS according to the HLH criteria.^5^ At the same time, fever, arthritis, oral ulcers, alopecia, high titer ANA, anti-double-stranded DNA, and low complement suggested a diagnosis of SLE according to Systemic Lupus International Collaborating Clinics classification criteria.^6^

The patient was treated with high dose of intravenous (IV) methylprednisolone 125 mg once a day for 3 days. At the end of high-dose methylprednisolone therapy, the patient’s fever resolved and skin rashes disappeared. The ferritin level decreased to 2026 ng/mL. Then, the drug was switched to prednisone 60 mg per os daily and hydroxychloroquine 200 mg per oral twice daily was added. The patient was discharged from the hospital and recommended to follow-up at the outpatient clinic.

## Discussion

The term “macrophage activation syndrome” was first used in a description of the disorder by Albert et al^7^ in 1992 and by Stephan et al in 1993.^8^ This terminology remains prevalent in the rheumatology literature, where as they often describe the similar phenomenon as secondary HLH in hematology and infectious literature.^9^ Nowadays, MAS is recognized as a secondary form of HLH.

MAS is a form of HLH that occurs primarily in patients with juvenile idiopathic arthritis or less commonly other rheumatologic disease. In 1991, Wong et al^10^ proposed a disease entity of acute lupus hemophagocytic syndrome, in which MAS occurs in association with the SLE flare, and corticosteroid therapy was effective in those cases. To date, only 26 cases of MAS at the onset of SLE are reported in the literature.^11^ We found other several cases of MAS due to SLE in the literature but these cases were related to SLE flare-up or complication. The features of MAS associated with SLE and active SLE are quite similar, so it is difficult to differentiate between these 2 conditions.

MAS is due to abnormal immune activation, which causes excessive inflammation and tissue destruction. Excessive inflammation is also due to lack of normal downregulation of activated macrophages, histiocytes, and lymphocytes. Macrophages become activated and secrete an excess amount of cytokines causing severe tissue destruction. Also, the function of NK cells to eliminate macrophages is lost. NK cells play a major role in maintaining immune responsiveness to noxious stimuli and are critical in prevention and control of autoimmune conditions. These cells also modulate the initial responses of antigen-presenting cells to incoming pathogens, thus diminishing the subsequent activation of antigen-specific T-cells. The loss of NK cell function results in excessive macrophage activity and elevated levels of interferon-γ and other cytokines. However, if the NK function is within normal limits, it should not exclude the diagnosis of HLH.^12^ Hemophagocytosis refers to the engulfment of host blood cells by macrophages. It is mediated through CD163 heme-scavenging receptor in macrophages.^13^ The proliferation of CD163+ hemophagocytic macrophages in marrow and lymphoid tissues is a central feature of the hemophagocytic disorder. It is characterized by the presence of red blood cells, platelets, or white blood cells within the cytoplasm of macrophages. It can also be seen on the biopsies of immune tissues (lymph nodes, spleen, and liver) or bone marrow. Although it is a marker of excess macrophage activation and supports the diagnosis of HLH, hemophagocytosis alone is not pathognomonic of diagnosis of HLH.^14^ Infiltration of bone marrow by active macrophages along with global clinical evaluation may distinguish HLH from other causes of hemophagocytosis.

The diagnosis of HLH is based on the following proposed HLH diagnostic criteria, 2004.^1^

1. Molecular identification of HLH associated gene mutation

Or 5 of the 8 criteria listed below are fulfilled:

1. Fever ⩾ 38.5°C
2. Splenomegaly
3. Cytopenias (affecting at least 2 of 3 cells in the peripheral blood, hemoglobin < 9 g/dL, platelets < 100 × 10^3^/mL, and neutrophils < 1 × 10^3^/mL)
4. Elevated triglycerides (fasting > 265 mg/dL) and/or low fibrinogen (<150 mg/dL)
5. Evidence of hemophagocytosis in bone marrow, liver, spleen, or lymph nodes
6. Low or absent NK cell activity
7. Ferritin > 500 ng/mL
8. Elevated sCD25 (α-chain of soluble IL-2 receptor)

Studies have indicated persistently elevated circulating level of multiple proinflammatory cytokines during symptomatic disease. It is currently believed that the symptoms generated by hypercytokinemia and hyperchemokinemia generated by uncontrolled activation of histiocytes and T-cells leads to progressive organ dysfunction that eventually leads to death in affected patients. These symptoms include fever, hyperlipidemia, endothelial activation/coagulopathy, hepatitis, central nervous system vasculitis, inflammatory lung disease with acute respiratory distress syndrome, and bone marrow hyperplasia or aplasia. Parodi et al^15^ mentioned that the strongest indicator to separate MAS from active SLE was hyperferritinemia, but they did not report whether ferritin level can be a predictor of disease severity or prognosis of the disease. Emmenegger et al^16^ showed that the absolute values did not strictly reflect the clinical severity from a comparison among individuals, but serial ferritin measurements in a single patient reliably reflected disease activity at least during the initial phase of MAS. Ferritin level above 10 000 mg/L is specific and sensitive for HLH.^5^ In patients without a significant medical history and new onset of febrile illness with highly elevated ferritin, HLH should be suspected.^17^ However, elevated ferritin is seen in the patients with adult-onset Still’s disease. Diagnosis of Still’s disease is based on daily spiking fever, evanescent rashes, and arthralgia, which can also be seen in HLH, but a distinction can be made on cytopenia, which is present in Still’s disease but not in HLH. Diagnostic confirmation through the demonstration of macrophage hemophagocytosis in the bone marrow may not be necessary in the presence of the typical clinical and laboratory features of the syndrome.^14^

Index of suspicion of MAS should be high when an infection is ruled out or inflammation persists and does not respond to treatment of an underlying condition. There are several therapeutic options available including biologic and nonbiologic agents. Effective initial therapy of HLH consists of the combination of immunosuppressive drugs targeting hyperactive T-cells and histiocytes. The mainstay of treatment is steroids. An IV methylprednisolone pulse therapy (30 mg/kg for 3 consecutive days) followed by 2 to 3 mg/kg/day is the most preferable. Several other drugs can be used in combination to reduce the dose of steroids. Hydroxychloroquine in combination with oral steroids is known to control the disease.^18^ Therapies that target activated macrophages/histiocytes (etoposide, steroids, and high-dose IV immunoglobulin) and activated T-cells (steroids, cyclosporine A, and anti-thymocyte globulin) are known to suppress HLH.^19^ The mortality rate is >90% in patients without treatment.^20^

## Conclusion

It is of paramount importance that fever, rash, and cytopenia can be clues to MAS and to look for secondary causes of MAS such as autoimmune disorder is essential as it affects management. Although it is very rare for a patient to have MAS as the initial presentation of SLE, the thorough history taking and timely immunological testing are essential because MAS due to SLE responds rapidly to steroids and immunosuppressants. Early diagnosis is crucial since mortality rates remain high for untreated cases.
